# Effects on lymph node size, staging and primary tumor histology on diagnostic accuracy of axillary lymph node aspirate of breast cancers

**DOI:** 10.1007/s10549-024-07533-1

**Published:** 2024-11-17

**Authors:** Joshua J. X. Li, Joanna K. M. Ng, Nikki K. Y. Hon, Ka Wun See, Julia Y. S. Tsang, Gary M. Tse

**Affiliations:** 1 Department of Pathology, School of Clinical Medicine, Queen Mary Hospital, The University of Hong Kong, Hong Kong Island, Hong Kong; 2 Department of Anatomical and Cellular Pathology, Prince of Wales Hospital, The Chinese University of Hong Kong, 1/F, Clinical Sciences Building, New Territories, Hong Kong; 3 Department of Pathology, Alice Ho Miu Ling Nethersole Hospital, New Territories, Hong Kong; 4 Department of Pathology, North District Hospital, New Territories, Hong Kong

**Keywords:** Breast, Cytology, Fine-needle aspiration, Lymph node, Metastasis

## Abstract

**Introduction:**

Fine-needle aspiration cytology is preferred for axillary lymph node metastasis with low costs and minimal risks. To improve diagnostic performance by incorporating clinical-radiological-pathological parameters, a large cohort pre-operative aspirates in were reviewed for parameters affecting adequacy rate and accuracy.

**Methods:**

Axillary nodal aspirates from three institutions with histologic correlation were retrieved. Case notes were reviewed for parameters pertaining to the primary tumor, nodal status, histologic and cytologic diagnoses.

**Results:**

Totally 1361 specimens were included. The risk of malignancy for C1–C5 categories were 53.39%, 27.45%, 70.97%, 83.33% and 88.00%, increasing to 75.86%, 94.59% and 99.28% for C3/C4/C5 categories excluding cases with neoadjuvant therapy. Node size (*p* < 0.001) and histologic grade (*p* = 0.003) of primary tumor positively correlated with specimen adequacy. Presence of in situ component trended towards inadequacy (*p* = 0.069). Lymph node size remained a strong predictor of concordant cytologic diagnosis (*p* < 0.001). A higher percentage of involved node (*p* = 0.006) and HER2 overexpressed breast cancers (*p* = 0.027) increased concordance. Cases with ≥ 4 (up to ≥ 10) positive nodes were more likely to be concordant (*p* = 0.009– < 0.001), with improvements of 8.27%–12.37%. For size, cut-offs of ≥ 5 and ≥ 10 mm were significant (*p* = 0.006– < 0.001).

**Conclusion:**

It is critical that clinical-radiological-pathological findings be interpreted together with cytology. Aspirates from smaller nodes are more likely to be non-informative, irrespective of the total number of suspicious nodes, or a high-grade primary. In axillae with less than 4 suspicious nodes and/or a target node of less than 5–10 mm, the diagnostic accuracy of aspiration cytology decreases and should be interpreted cautiously.

## Introduction

In the assessment of axillary lymph node status for breast cancers, fine-needle aspiration cytology is the preferred diagnostic modality with a low cost and minimal risk of complications [1]. Although it has been widely accepted that lymph node aspiration cytology, for detection of metastasis, is highly specific with slight limitations on sensitivity [2], the application of aspiration cytology for breast cancer metastasis is different. Clinical, radiological and histopathological findings of the lymph node and primary tumor are usually available before aspiration is performed, unlike in the assessment of lymphadenopathies of unknown nature [3, 4]. In this study, a large cohort of axillary lymph node aspirates in pre-operative assessment for breast cancer patients were reviewed for lymph node and histopathological parameters affecting the adequacy rate and accuracy of cytologic diagnosis.

## Methods

**Fig. 1:**
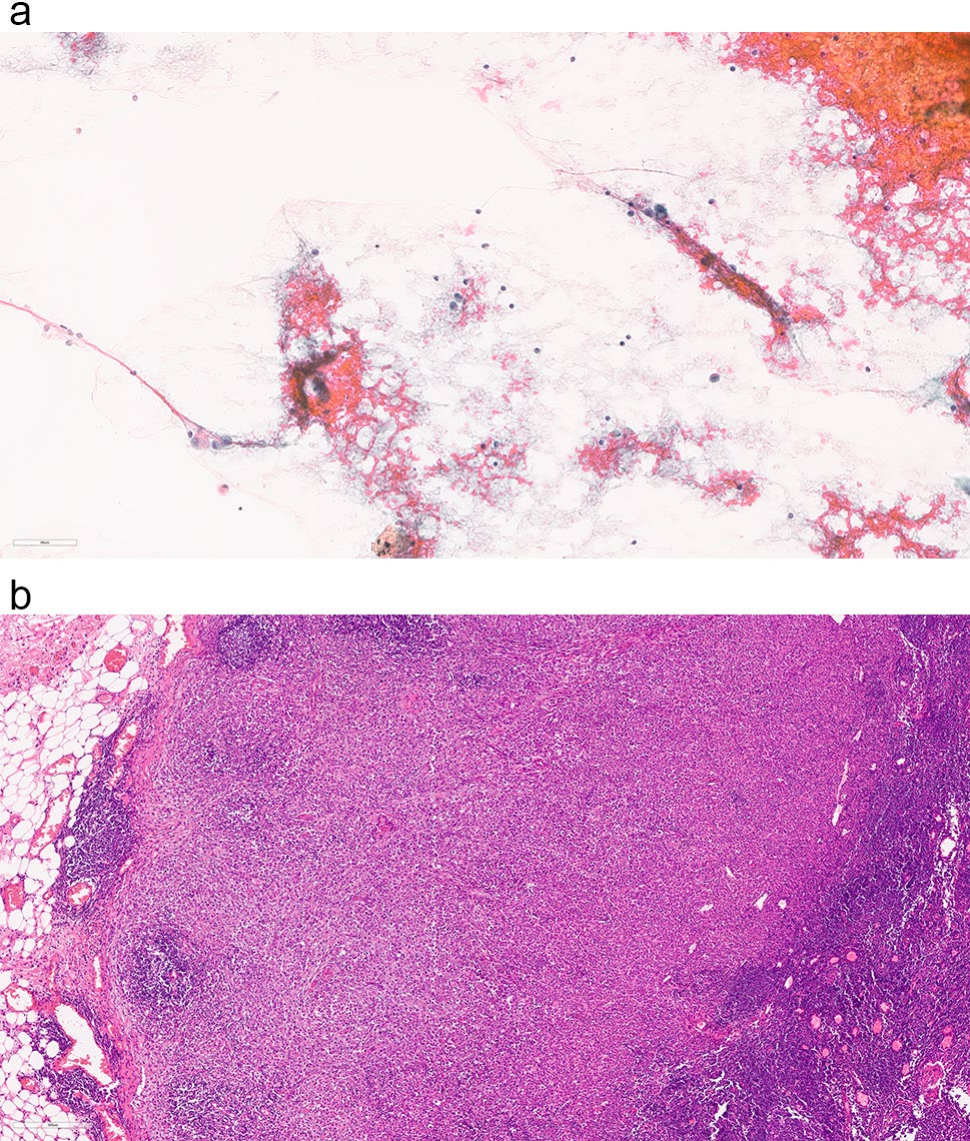
Axillary lymph node aspirate and corresponding histology, **a** scattered irregular atypical cells, the cytologic diagnosis was C3 (atypia), 400 × magnification, **b** Axillary dissection showed metastatic lobular carcinoma, 100 × magnification

Computerized searches of the three involved institutions for axillary node fine-needle aspirate cytology specimens were performed (Alice Ho Miu Ling Nethersole Hospital and North District Hospital: from the year 2000 to 2022; Prince of Wales Hospital from the year 1997 to 2022). Cytologic diagnoses were reclassified into a five-tiered system—inadequate/unsatisfactory (C1), benign (C2), atypia (C3), suspicious for malignancy (C4) and malignant (C5). Case notes and pathology reports of each patient was reviewed, and those with corresponding sentinel lymph node core or excision biopsy and/or axillary lymph node dissection performed were included (Fig. 1). Radiology reports, pathology reports and clinical notes were reviewed in sequence for the greatest diameter of the target lymph node (i.e., clinical dimensions taken when radiological and pathological sizing were not present). Histological parameters of the primary breast tumor, including histological type, histological grade and presence of in situ components were recorded from corresponding breast biopsy and/or surgical excision reports. Case notes, cytology reports and/or slides with a false positive result (no histologic evidence of lymph node metastasis but a cytologic diagnosis of C3, C4 or C5) were further reviewed for possible causes including but not limited to interval neoadjuvant treatment, other neoplasms involving the axilla and cytologic interpretation.

Statistical analysis was performed using SPSS (version 26.0). The chi-squared test and t-test were used to compare lymph node parameters including the size of the targeted lymph node, number of positive and total number of lymph nodes excised, and histological parameters of the primary tumor. Comparisons were made between inadequate (C1) and adequate (C2-C5) specimens, and between concordant and discordant cases at binary cutoffs considering atypia or higher-grade cytologic diagnoses (C3 +) or suspicious or higher-grade cytologic diagnoses (C4 +) as positive and excluding C1 specimens. A *p*-value of < 0.05 was considered as significant. The study was approved by The Joint Chinese University of Hong Kong – New Territories East Cluster Clinical Research Ethics Committee.

**Table 1:** Demographics of the cohort

Total number of cases	1361
Laterality	
Left	697
Right	664
Histologic type	
Apocrine carcinoma	1
Ductal carcinoma in situ	68
Invasive ductal carcinoma	1094
Invasive lobular carcinoma	43
Lobular carcinoma in situ	1
Mucinous carcinoma	27
Medullary carcinoma	3
Metaplastic carcinoma	13
Mixed	27
Neuroendocrine carcinoma	1
Papillary carcinoma	14
Phyllodes tumor	2
Sarcoma	1
Tubular carcinoma	2
Invasive carcinoma/malignancy, not-specified	64
Histological grade	
Grade 1	166
Grade 2	550
Grade 3	463
Surrogate molecular subtype	
Luminal (A/B)	164
HER2 overexpressed	868
Triple negative	198

## Results

A total of 1361 fine-needle aspiration cytology specimens were included, with 697 from the left and 664 from the right axilla. The primary breast tumor was invasive ductal carcinoma (invasive breast carcinoma, no special type) (*n* = 1094/1361 80.38%), followed by ductal carcinoma in situ (*n* = 68/1361, 5.00%) and invasive lobular carcinoma (*n* = 43/1361, 3.16%) (Table 1). The risk of malignancy for the C1 to C5 categories were 53.39%, 27.45%, 70.97%, 83.33% and 88.00%, increasing to 75.86%, 94.59% and 99.28% for the C3, C4 and C5 categories when cases with neoadjuvant therapy were excluded (Table 2).

**Table 2:** Risk of malignancy (ROM) in each cytologic category

	Total ROM	ROM excluding cases with neoadjuvant therapy
C1	53.39% (71/133)	
C2	27.45% (137/499)	
C3	70.97% (44/62)	75.86% (44/58)
C4	83.33% (35/42)	94.59% (35/37)
C5	88.00% (550/625)	99.28% (550/554)

Comparison of inadequate and adequate specimens demonstrated that the greatest diameter of the target lymph node (i.e., size) (*p* < 0.001) and the histologic grade (*p* = 0.003) of the primary tumor were positively correlated with an adequate cytologic diagnosis. The presence of an in situ component in the primary breast tumor showed a trend towards an inadequate cytologic diagnosis (*p* = 0.069). The number of positive lymph nodes, total number of lymph nodes, percentage of positive nodes and surrogate molecular subtype did not correlate with specimen adequacy (*p* > 0.05) (Table 3).

**Table 3:** Correlation between lymph node and primary tumor parameters with specimen adequacy

	Inadequate (C1)	Adequate (C2-C5)	*p*-value
No. of positive lymph nodes	2.58	3.25	0.339
Total no. of lymph nodes	14.25	15.22	0.146
% of positive lymph nodes	18.19%	20.57%	0.197
Greatest diameter (mm)	9.05	13.15	< 0.001
Histologic grade			
Grade I	29	137	
Grade II	48	502	
Grade III	42	421	0.003
In situ component			
Present	91	741	
Absent	42	487	0.069
Surrogate molecular subtype			
Luminal	16	148	
HER2 overexpressed	88	780	
Triple negative	13	185	0.301

The greatest diameter of the target lymph node remained a strong predictor of concordance in two cytologic diagnosis categorical cut-offs, with a p-value of less than 0.001. A higher percentage of involved lymph node (*p* = 0.006) and HER2 overexpressed breast cancers (*p* = 0.027) were associated greater concordance rates, considering C4 and C5 as a positive diagnostic result. Other parameters compared did not show statistical significance, and except for lymph node diameter, no other parameter was significantly correlated with concordance when cytologic diagnoses of C3 or above were taken as positive (Table 4).

**Table 4:** Comparison of diagnostic concordance with lymph node and primary tumor parameters a) considering C3 (atypia) or b) C4 (suspicious) or above as positive

	(a) Concordant	Discordant	*p*-value	(b) Concordant	Discordant	*p*-value
No. of positive lymph nodes	3.68	2.20	0.346	3.57	3.03	0.083
Total no. of lymph nodes	15.32	14.07	0.080	15.17	15.06	0.870
% of positive lymph nodes	21.70%	23.95%	0.346	21.03%	27.10%	0.006
Greatest diameter (mm)	13.20	10.02	0.001	13.25	10.46	0.001
Histologic grade						
Grade I	104	24		102	26	
Grade II	410	69		400	79	
Grade III	357	46	0.094	344	59	0.310
In situ component						
Present	600	100		579	121	
Absent	391	57	0.452	384	64	0.177
Surrogate molecular subtype						
Luminal (A/B)	118	14		117	15	
HER2 overexpressed	630	115		606	139	
Triple negative	153	19	0.153	151	21	0.027

^*^Cases with neoadjuvant treatment excluded

The concordance rates for cases with at least one positive lymph node compared to all lymph nodes negative were lower (C3 + : 82.11% vs. 94.76%; C4 + : 76.63% vs. 98.43%). However, the difference in concordance rates were reversed at higher cut-offs. Aspirates in cases with at least 4 or more (and at least 10 or more) positive lymph nodes were more likely to be concordant (*p* = 0.009–< 0.001), with differences in concordance rates at 8.27% to 12.37%. As for the greatest diameter of the target lymph node, a greater diameter was associated with higher concordance rates consistently, with significant differences at cut-offs of ≥ 5 mm (*p* = 0.001 and *p* = 0.006) and ≥ 10 mm (p < 0.001) and statistical trends at ≥ 20 mm (*p* = 0.090 and *p* = 0.051) (Table 5).

**Table 5:** Diagnostic concordance according to lymph node parameters (a) considering C3 (atypia) or (b) C4 (suspicious) or above as positive at different cut-offs

	(a) Concordant	Discordant	Concordance rate	*p*-value	(b) Concordant	Discordant	*p*-value	Concordance rate
No. of positive nodes			(Yes/no)					(Yes/No)
≥ 10 (Yes/no)	114/877	3/154	97.44%/85.06%	< 0.001	108/855	9/176	0.009	92.31%/82.93%
≥ 4 (Yes/no)	333/658	29/128	91.99%/83.72%	< 0.001	314/649	48/137	0.074	86.74%/82.57%
≥ 3 (Yes/no)	401/590	51/106	88.72%/84.77%	0.057	378/585	74/111	0.849	83.63%/84.05%
≥ 2 (Yes/no)	500/491	81/76	86.06%/86.60%	0.791	466/497	115/70	0.001	80.21%/87.65%
≥ 1 (Yes/no)	629/362	137/20	82.11%/94.76%	< 0.001	587/376	179/6	< 0.001	76.63%/98.43%
Greatest diameter								
≥ 20 mm (Yes/no)	122/537	11/85	91.73%/86.33%	0.090	119/514	14/108	0.051	89.47%/82.64%
≥ 10 mm (Yes/no)	415/244	40/56	91.21%/81.33%	< 0.001	398/235	57/65	< 0.001	87.47%/78.33%
≥ 5 mm (Yes/no)	576/83	72/24	88.89%/77.57%	0.001	553/80	95/27	0.006	85.34%/74.77%

^*^Cases with neoadjuvant treatment excluded

## Discussion

Axillary lymph node metastasis is a strong prognostic indicator in breast cancer [5]. The treatment options are axillary lymph node dissection, radiotherapy and/or combinations with other systemic therapy [6]. Axillary recurrence is associated with significant morbidity and mortality [7, 8], with nearly half of the patients further developing distant metastases [9]. On the other hand, axillary dissection and regional radiotherapy are associated with risks of functional morbidities such as lymphedema, paresthesia and other forms of impairment [10]. Both overtreatment and undertreatment should be avoided. Accurate pre-operative diagnosis is necessary in preventing inadequate treatment.

The triple assessment also applies to the axilla [11]. Fine-needle aspiration is highly suited for breast and axillary lymph node biopsy [12]. With the superficial and palpable location of the axillary nodes, the need of puncture in multiple directions to adequately sample a lymph node, and a relatively favorable cost and complication profile of fine-needle aspiration over core biopsy [3, 13], axillary lymph node aspiration is often used in primary tissue diagnosis for axillary nodal status. Despite extensive literature highlighting the specificity of axillary lymph node aspiration cytology [14], two caveats must be addressed – the diagnostic accuracy of cytology depends on how the diagnostic categories are attributed (in particular the atypia group) [2], and cytology results are not interpreted in isolation but with clinical and radiological findings. In this study, the histologic correlation of axillary lymph node aspiration from a cohort including multiple centers and collected through an extended period, were reviewed in correlation with clinical and radiological parameters to detail the diagnostic performance and possible effects of clinical and radiological features.

In line with the literature, the sensitivity of axillary lymph node aspiration cytology was modest, with a 27.45% ROM, equivalent to the false negative rate [14]. The ROMs of the C3 to C5 categories were initially slightly low, ranging from 70% to less than 90%, but when cases with neoadjuvant therapy were excluded, the ROMs of C4 and C5 categories improved to 94.50% and 99.28%. There were 16, 2, and 4 false positives in the C3, C4 and C5 categories, respectively. Review of the cases found one with primary sarcoma involving the axillary region, and one case with ductal carcinoma in situ involving the axillary tail, without invasion nor lymph node involvement. The remaining 18 were all attributed to interpretative errors (Table 2).

Inadequate aspiration necessitates repeat biopsy and leads to delay in management and increased resources consumption [15, 16]. It should be noted that the risk of malignancy for inadequate specimens is significantly higher than C2 and cannot be considered as a “provisionally” benign diagnosis. There were also 68 cases of ductal carcinoma in situ included for analysis, of which 10 had histologic evidence of lymph node metastasis. Ductal carcinoma in situ with metastasis is a well reported phenomenon that is largely attributed to sampling error and minute undetected or undetectable invasive foci [17], as such the cases were included for analysis. Two inadequate diagnosis that had positive lymph node histology were also present in the group of ductal carcinoma in situ, indicating that even in low-risk cases, inadequate aspirates necessitate further workup.

Excisional or sentinel node biopsy may be preferable when pre-operative diagnostic yield is expected to be low, such as in cases of classical lobular carcinoma (18). Low-grade histology and small lymph node size were associated with an increased inadequacy rate in the current cohort. Omission of substitution of fine-needle aspiration by other biopsy modalities such be considered for these cases. Of note, only the size of the target, node demonstrated correlation, whereas the total number of positive nodes does not affect adequacy rate, suggesting that targeting the largest node is necessary even when there are multiple suspicious lymph nodes.

The limitations of the study includes the heterogeneity of cases over a long collection period, with different clinical guidelines and protocols adopted, and its retrospective design. Clinically and radiologically low-risk cases, particularly those with benign or inadequate aspirates, may not be subjected to further axillary lymph node dissection or even core or excisional biopsy. On the other hand, patients with advanced disease also may not be treated surgically and thus not captured in the cohort. The requirement of histological correlation skewed the composition of breast cancer cases and would not match the composition histological or molecular typing in the breast cancers of the general population.

As for clinical and radiological parameters affecting the diagnostic performance of lymph node aspiration, lymph node size was identified as a consistent predictor of concordance regardless of cut-off adopted in cytologic diagnostic category. Cases with higher percentage of involved lymph node and HER2 overexpressed breast cancers showed higher concordance at the C4 or above cut-off but not for C3 or above. Further analysis pertaining to these significant parameters showed that for size, lymph nodes with greatest dimension of greater or equal to 5 mm and 10 mm statistically significantly improved diagnostic accuracy, and a trend was observed for the cut-off at least 20 mm. The concordance rate also increased with the number of positive nodes, reaching statistical significance at the threshold of greater or equal to 4 positive nodes. These figures can serve as a reference in deciding whether fine-needle aspiration should be proceeded, or in cases where there is discrepancy between cytologic diagnosis, clinical impression and radiological findings, further investigation is needed for clarification.

## Conclusion

Fine-needle aspiration is specific but suffers from a lower sensitivity in detecting lymph node metastasis. As such, it is critical that clinical and radiological findings be examined together with cytologic results. Smaller lymph nodes are more likely to be non-informative on aspiration cytology, irrespective of whether other suspicious lymph nodes are present, or if the primary lesion if of a high histologic grade. In axillae with less than 4 suspicious lymph nodes and/or a target lymph node of less than 5–10 mm, the diagnostic accuracy of axillary lymph node aspiration decreases and should be interpreted with caution.

## Data Availability

The authors declare all data generated are available in the manuscript.
